# The Superficial Stromal Scar Formation Mechanism in Keratoconus: A Study Using Laser Scanning In Vivo Confocal Microscopy

**DOI:** 10.1155/2016/7092938

**Published:** 2016-01-18

**Authors:** Peng Song, Shuting Wang, Peicheng Zhang, Wenjie Sui, Yangyang Zhang, Ting Liu, Hua Gao

**Affiliations:** Shandong Eye Hospital, Shandong Eye Institute, Shandong Academy of Medical Sciences, Jinan 250021, China

## Abstract

To investigate the mechanism of superficial stromal scarring in advanced keratoconus using confocal microscopy, the keratocyte density, distribution, micromorphology of corneal stroma, and SNP in three groups were observed. Eight corneal buttons of advanced keratoconus were examined by immunohistochemistry. The keratocyte densities in the sub-Bowman's stroma, anterior stroma, and posterior stroma and the mean SNP density were significantly different among the three groups. In the mild-to-moderate keratoconus group, activated keratocyte nuclei and comparatively highly reflective ECM were seen in the sub-Bowman's stroma, while fibrotic structures with comparatively high reflection were visible in the anterior stroma in advanced keratoconus. The alternating dark and light bands in the anterior stroma of the mild-to-moderate keratoconus group showed great variability in width and direction. The wide bands were localized mostly in the posterior stroma that corresponded to the Vogt striae in keratoconus and involved the anterior stroma only in advanced keratoconus. Histopathologically, high immunogenicity of *α*-SMA, vimentin, and FAP was expressed in the region of superficial stromal scarring. In vivo confocal microscopy revealed microstructural changes in the keratoconic cone. The activation of superficial keratocytes and abnormal remodeling of ECM may both play a key role in the superficial stromal scar formation in advanced keratoconus.

## 1. Introduction

Keratoconus is an ectatic corneal disorder characterized by progressive, central, or paracentral corneal thinning, which results in apical corneal protrusion, irregular astigmatism, superficial scar formation, and significantly decreased vision [1, 2]. Previous pathologic studies concluded that underlying abnormalities in the stromal repair and reactive species-linked activities and the interaction between these phenomena were implicated in the development of keratoconus [3, 4].

Corneal scarring is a commonly occurring consequence of several forms of trauma, wounds, chemical burns, infections, and refractive surgery; however, keratoconic patients do not have corneal trauma, epithelial defects, or any inflammatory diseases. Instead, altered expression of wound healing and stress-related proteins has been found in keratoconic corneas [5]. Previous reports have demonstrated that regions with stromal scarring and fibrotic deposits have comparatively high expressions of laminin-5, proteoglycans, fibrillin-1, tenascin-C, and types IV, VII, and VIII collagen [5–7]. Little information is available regarding this superficial scar formation although there have been a few confocal microscopic or histopathological findings [3, 8, 9]. A histopathological examination of patients with advanced keratoconus showed that the posterior stroma remained undisturbed in keratoconus, but tissue (lamellae) debris was detected in the anterior stroma [3]. Moreover, confocal microscopy revealed significant microstructural alterations and a decrease in keratocytes in the anterior and posterior stroma in patients with advanced keratoconus [9, 10].

Studies of superficial stromal scar formation ex vivo in keratoconus are subject to certain restrictions. The managements of mild-to-moderate keratoconus include rigid gas permeable contact lens fitting, corneal collagen crosslinking, and intrastromal corneal ring segments [11, 12]. As keratoplasty is not considered as a treatment option for mild-to-moderate keratoconus, it is difficult to collect mild-to-moderate keratoconus buttons for investigation ex vivo. Confocal microscopy allows a noninvasive view of the living human cornea at magnifications of up to 800x. We hypothesized that microstructural abnormalities may occur in the stroma and lamellae during corneal protrusion in mild-to-moderate keratoconus and thus contribute to the progression of keratoconus and superficial scar formation. The aim of the present study was to investigate the corneal microstructural and histopathological changes in keratoconus, which may be the important proofs of mechanical failure, and relate these to the superficial stromal scar in keratoconus.

## 2. Materials and Methods

### 2.1. Subjects and Design

This prospective and cross-sectional comparative study was approved by the Ethics Committee of the Shandong Eye Institute and followed the tenets of the Declaration of Helsinki. Written informed consent was obtained from each participant.

All patients were diagnosed with keratoconus by clinical examinations, which included slit-lamp biomicroscopy, keratometry, A-scan ultrasonography, corneal topography, and in vivo laser scanning confocal microscopy. A positive diagnosis of keratoconus was based on the presence of at least one keratoconic feature (centric or eccentric corneal stromal thinning, Vogt striae, Fleischer ring, or Munson's sign) and topographic findings (an increased area of corneal power surrounded by concentric areas of decreasing power, inferior-superior power asymmetry, an inferior-superior dioptric asymmetry difference greater than 1.4 diopters, and skewing of the steepest radial axes above and below the horizontal meridian). Keratoconic eyes were divided into two groups based on the Keratoconus Severity Score (KSS) and modified Krumeich classification of keratoconus (Table 1) [12, 13]. The control group consisted of healthy individuals without risk factors for keratoconus. The diagnosis of keratoconus was made by Peng Song and Hua Gao, and the two doctors' agreement rate was 100%.

The exclusion criteria were as follows: a history of ocular trauma, any previous ocular surgery, the use of any systemic or ocular medications, coexisting corneal pathology, keratoconic eyes with acute corneal hydrops or perforation, contact lens wear, or the presence of systemic disease that may affect the cornea.

### 2.2. In Vivo Confocal Microscopy

The primary laser scanning in vivo confocal microscope was the Heidelberg Retina Tomograph HRT-2 (Heidelberg Engineering GmbH, Heidelberg, Germany); the addition of the Rostock Cornea Module (RCM) converted it into an in vivo confocal microscope of the eye surface. Before the measurement, 1 drop of topical anesthetic (0.5% proparacaine, Alcon, Fort Worth, TX) and 1 drop of carbomer eye gel (Bausch & Lomb, Rochester, New York, NY) were applied. A sterile and disposable polymethylmethacrylate cap was placed onto the head of the objective. The patient's chin was placed on the related part of the apparatus. The objective was zoomed onto the central cornea using the joystick. To ensure that only measurements in the central cornea were taken, the patient was instructed to fixate on a small red light with the contralateral eye. Centered and pressure-free contact of the HRT-2 with the cornea was monitored optically using a swivel color camera. The full thickness of the central cornea or the center of the keratoconic cone (within the central 2 mm diameter) was scanned using the “section mode” of the device. This mode enables instantaneous imaging of a single area of the cornea at a desired depth. All the confocal microscopic examinations were performed by the same investigator (Shuting Wang).

The following corneal layers were subjected to in vivo confocal microscopy: (1) the subbasal nerve plexus (SNP); (2) sub-Bowman's stroma (the layers of the stroma below Bowman's membrane); (3) the anterior stroma (the layers comprising one-third of the stroma); and (4) the posterior stroma (the layers comprising two-thirds of the stroma). The keratocytes were counted by a semiautomated cell counter. The focused layers of the anteroposterior keratocytes were recorded. Three randomly chosen images of three desired stromal layers per subject were analyzed, statistically compared, and averaged. Within the counting frame, the truncated cells of the left and lower edges were considered. The results were extrapolated up to an area of 1 mm^2^. The SNP density was evaluated using NeuronJ, a free semiautomatic image analysis program, and a plug-in to the program, ImageJ (http://www.imagescience.org/meijering/software/neuronj/; accessed November 2012). Three randomly chosen images of the SNP per subject were analyzed, statistically compared, and averaged. The measurement of keratocyte density and SNP density was performed by two masked examiners (Peng Song and Peicheng Zhang).

### 2.3. Immunohistochemistry

The significance of FAP and vimentin was the major morphological characteristic of the fibroblasts, and the expression of vimentin corresponded to the expression of FAP. At least three 4 mm sections were obtained from the paraffin-embedded corneal buttons. The antigens were recovered by microwaving for 15 min in an EDTA solution. Endogenous peroxidase activity was quenched by incubating the sections in 3% hydrogen peroxide for 5 min. Normal goat serum was used to block nonspecific staining. The sections were subsequently incubated with mouse antifibroblast activation protein (Abcam, Hong Kong, China), mouse anti-Vimentin (Abcam), and mouse anti-*α*-smooth muscle actin (Maxim, Fujian, China) for 60 min at 37°C, a reinforcing agent (Maxim) for 15 min at 37°C, and HRP-conjugated goat anti-mouse IgG for 30 min at 37°C. Peroxidase activity was visualized by incubating the sections in a diaminobenzidine (DAB) solution (Maxim). Negative controls were performed in the absence of primary antibodies. Finally, the samples were mounted and examined with a microscope (Nikon Eclipse E800, Nikon, Tokyo, Japan).

### 2.4. Statistical Analysis

Statistical analyses were performed using SPSS 19.0 (IBM). The Kolmogorov-Smirnov test was used to evaluate the distribution characteristics of the variables. The one-way ANOVA test and Kruskal-Wallis test were used to compare the variables between the groups. The Bonferroni method was used for adjustment so as to make multiple comparisons. Differences were considered statistically significant for *P* values less than 0.05.

## 3. Results

### 3.1. Demographic Profiles and Baseline Clinical Characteristics

Seventy eyes of 44 patients with keratoconus were selected from patients who were diagnosed with keratoconus at the Shandong Eye Institute from March 2012 to October 2014 and divided into groups of mild-to-moderate keratoconus (35 eyes) and advanced keratoconus (35 eyes). The control group comprised 20 healthy eyes of 20 volunteers recruited from the hospital staff and their relatives. The demographic and topographic features of the three groups are shown in Table 2. No statistically significant difference was observed in the age and gender distribution among the three groups.

**Figure 1:**
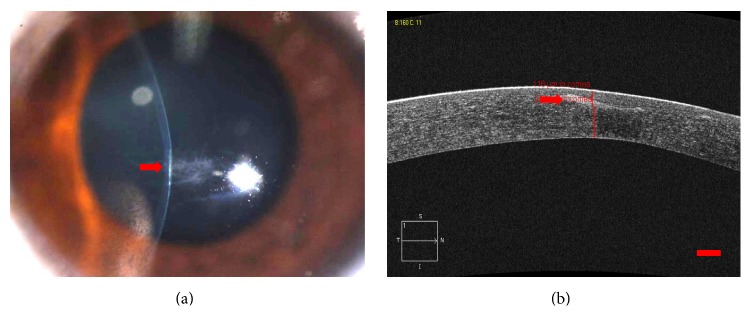
Advanced keratoconus. (a) The appearance of superficial stromal scarring during the slit-lamp examination with a narrow-band light. (b) Stromal scarring was visible in the superficial stroma during the OCT examination. Scale bar: 200 *μ*m.

Using slit-lamp microscopy, Vogt striae were found in 6 (17.1%) of 35 eyes with mild-to-moderate keratoconus and 29 (82.9%) of 35 eyes with advanced keratoconus. The Fleischer ring was found in 13 eyes (37.1%) with mild-to-moderate keratoconus and 33 eyes (94.3%) with advanced keratoconus. Superficial stromal scarring was detected in 8 eyes (22.9%) with advanced keratoconus (Figure 1) but not in any eye with mild-to-moderate keratoconus. None of the above was observed in the controls.

### 3.2. Clinical Examination with In Vivo Scanning Confocal Microscopy

**Figure 2:**
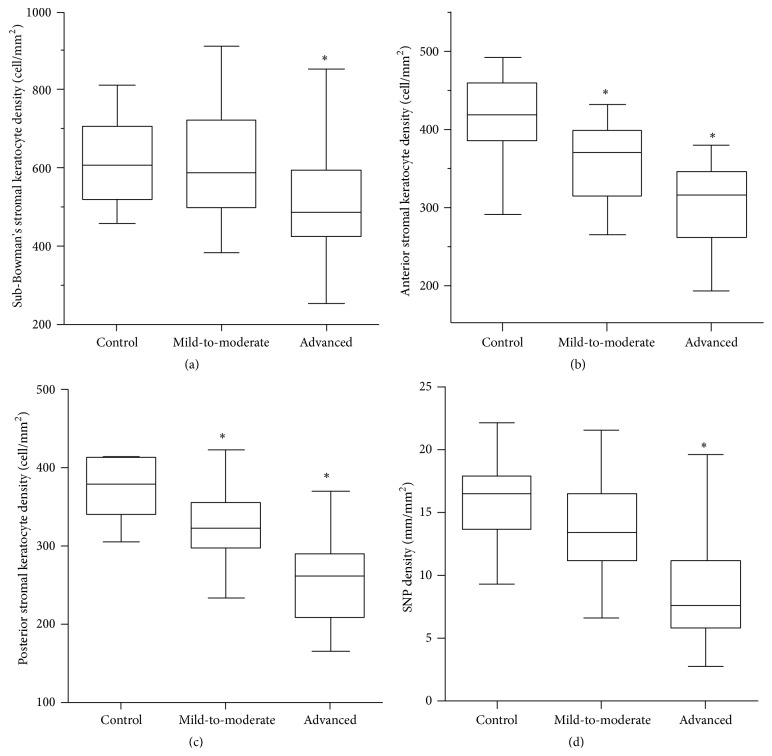
Box plots of keratocytes and nerve plexus densities measured by in vivo confocal microscopy. (a) Sub-Bowman's stromal keratocyte density in advanced keratoconus, (b) the anterior stromal keratocyte density in mild-to-moderate and advanced keratoconus, (c) the posterior stromal keratocyte density in mild-to-moderate and advanced keratoconus, and (d) the SNP density in advanced keratoconus were significantly lower than in the control subjects (∗. all *P* < 0.05).

The confocal microscopy findings are shown in Table 3. The mean densities of sub-Bowman's stromal keratocytes, anterior stromal keratocytes, posterior stromal keratocytes, and subbasal nerve plexus were significantly different among the three groups (*P* = 0.008, *P* < 0.001, *P* < 0.001, and *P* < 0.001, resp.). The mean sub-Bowman's stromal keratocyte density was significantly lower in the advanced keratoconus group but not in the mild-to-moderate keratoconus group when compared with the control group (*P* = 0.036 and *P* < 0.001, resp.) (Figure 2(a)). The mean anterior stromal keratocyte density was significantly lower in both the mild-to-moderate keratoconus group and the advanced keratoconus group when compared with the control group (*P* = 0.002 and *P* < 0.001, resp.) (Figure 2(b)). The mean posterior stromal keratocyte density was significantly lower in both the mild-to-moderate keratoconus group and the advanced keratoconus group when compared with the control group (*P* = 0.003 and *P* < 0.001, resp.) (Figure 2(c)). The mean subbasal nerve plexus density was significantly lower in the advanced keratoconus group but not in the mild-to-moderate keratoconus group when compared with the control group (*P* < 0.001 and *P* = 0.095, resp.) (Figure 2(d)).

**Figure 3:**
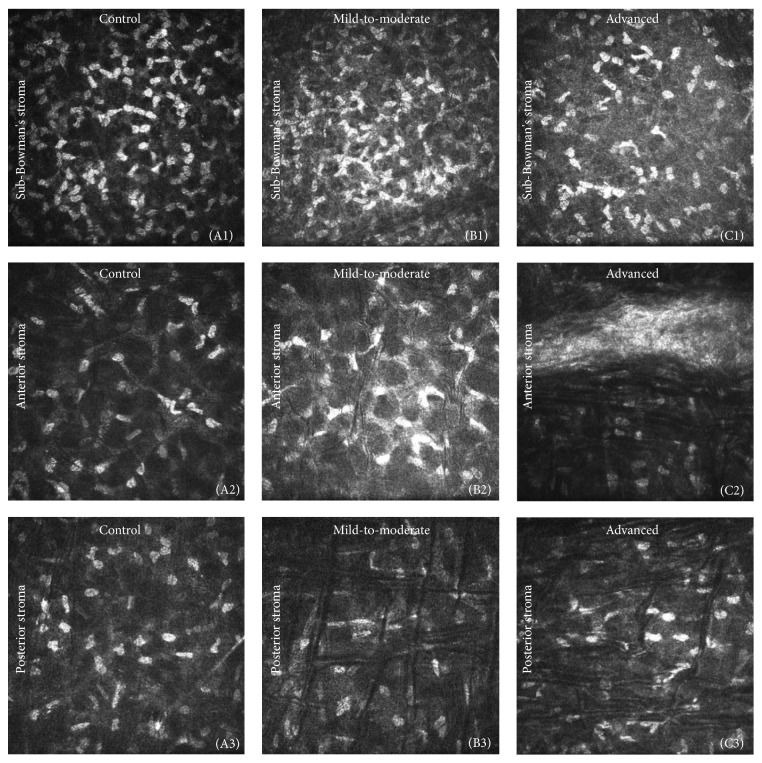
In vivo confocal microscopy images of the stroma in sub-Bowman's (top row) and anterior (middle row) and posterior stroma (bottom row) in the healthy controls, the mild-to-moderate keratoconus group, and the advanced keratoconus group, respectively. Image size: 400 × 400 *μ*m. (A1) Normal keratocytes. Clearly demarcated, highly reflective, oval keratocyte nuclei. (B1) Activated nuclei with variable morphology that are unidentifiable individually. Highly reflective extracellular matrix (ECM). (C1) Lower keratocyte density and higher ECM reflectivity than (A1) and (B1). Hyperreflective keratocytes with activated nuclei and fewer cytoplasmic processes. (A2) Normal keratocytes. Lower keratocyte density than sub-Bowman's stromal keratocyte density. (B2) Activated keratocytes, thin and varied-orientation bands, and highly reflective ECM. (C2) Wider and regular bands and hyperreflective deposits were visible. (A3) Normal keratocytes in the posterior stroma. (B3) Wider and orthorhombic bands, rare keratocyte nuclei located in the bands. (C3) The number of bands was greater than those in the mild-to-moderate keratoconus group, and the arrangement of striae was more disordered.

Confocal microscopic images revealed the extent of the variation in density and micromorphology of the keratocytes and SNP in the three groups (Figure 3). The keratocyte density in sub-Bowman's stroma was higher than the anterior and posterior stroma in all the groups. There was no significant difference in sub-Bowman's stromal keratocyte density between the controls and the mild-to-moderate keratoconus group, but an obvious decrease of keratocytes in the advanced keratoconus group (Figure 3(C1)) was visible compared with the other groups (Figures 3(A1) and 3(B1)). Highly reflective, sharply demarcated, and closely arranged cell nuclei of keratocytes were visualized in sub-Bowman's stroma in the controls, and the cytoplasm of this fibroblast subpopulation and the collagen fibers produced by them were not detectable (Figure 3(A1)). In the mild-to-moderate keratoconus group, keratocytes with activated nuclei and cytoplasmic processes produced a network appearance in sub-Bowman's stroma. The activated nuclei were branched and less readily identifiable individually, and the cytoplasm processes were also more than normal keratocytes (Figure 3(B1)). Moreover, the keratocytes had fewer cytoplasm processes and activated nuclei had reduced branches in the advanced keratoconus group (Figure 3(C1)).

In the anterior stroma, an obvious decrease in keratocytes was visible in the mild-to-moderate and advanced keratoconus groups when compared with the controls (Figures 3(A2), 3(B2), and 3(C2)). The anterior keratocytes in the mild-to-moderate keratoconus group had the same appearance as sub-Bowman's keratocytes, and a lower keratocyte density and more highly reflective cytoplasm and extracellular matrix (ECM) were detected compared with the controls (Figure 3(B2)). In the anterior stroma of the advanced keratoconus group, there were hyperreflective deposits, which corresponded with the superficial corneal scarring. In this zone, the keratocyte nuclei were not clearly visible (Figure 3(C2)).

In the posterior stroma, a decreasing trend of keratocyte density was found (Figures 3(A3), 3(B3), and 3(C3)). Alternating dark and light bands were visible in the anterior and posterior stroma of the eyes with keratoconus. In the anterior stroma, wider and more regular bands were presented in the advanced keratoconus group than in the mild-to-moderate keratoconus group (Figures 3(B2) and 3(C2)). The number and variation of striae in the posterior stroma were greater in the advanced keratoconus group than in the mild-to-moderate keratoconus group (Figures 3(B3) and 3(C3)). The direction of the bands was variable among the patients, mostly in an approximately vertical direction in the posterior stroma.

**Figure 4:**
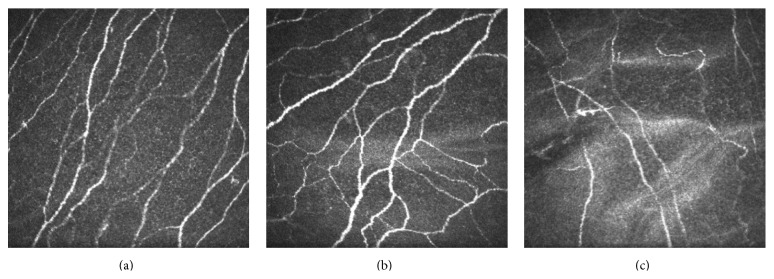
Subbasal nerve plexus images obtained by scanning slit confocal microscopy. Image size: 400 × 400 *μ*m. (a) Normal nerves in the controls. Parallel nerve fiber bundles with bunches. (b) The SNP in mild-to-moderate keratoconus. There was a tortuous network of nerve fiber bundles. (c) The SNP in advanced keratoconus. Short, interrupted, and disbranched nerve fibers were visible.

Examples of confocal microscopy images of the SNP in the control, mild-to-moderate keratoconus, and advanced keratoconus groups are shown in Figure 4. The normal nerve fibers in the controls mostly had thick, usually stretched, highly reflective structures, and some nerves were ramified into several fine branchlets (Figure 4(a)). In the patients with mild-to-moderate keratoconus, the nerve fibers had a thickened, prominent appearance, such as excessive branching, curling, and even closed loops, but had no significant difference in SNP density compared with the controls (Figure 4(b)). The SNP density in the advanced keratoconus group was significantly lower than that of the control and mild-to-moderate keratoconus groups. The nerve fibers appeared short, interrupted, and disbranched (Figure 4(c)).

### 3.3. Immunohistochemistry

**Figure 5:**
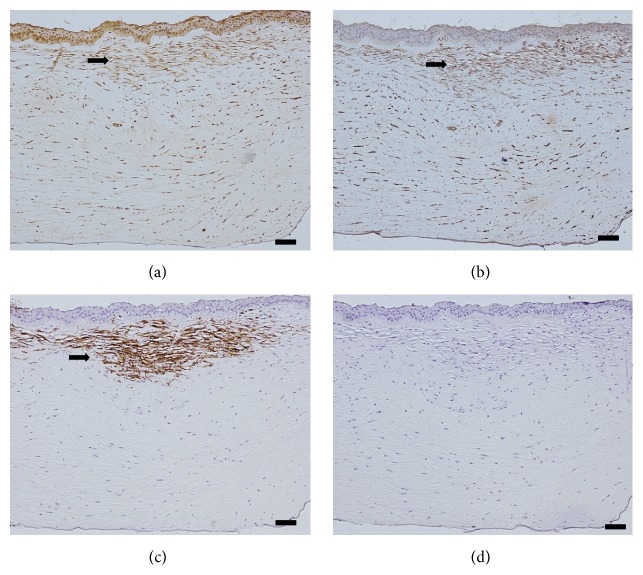
A patient with advanced keratoconus. (a) High FAP expression in the superficial stroma (black arrow) and decentralized expression in the posterior stroma. (b) Vimentin expression in the superficial stroma (black arrow) and decentralized expression in the posterior stroma. (c) *α*-SMA expression in the superficial stroma (black arrow). (d) Negative controls in the absence of primary antibodies. Scale bar: 100 *μ*m.

**Figure 6:**
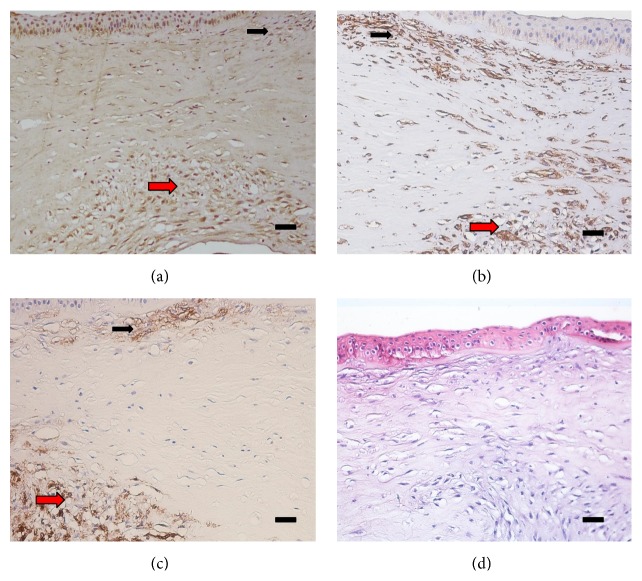
A patient with advanced keratoconus suffered from superficial corneal scarring and posterior corneal scarring due to rupture of Descemet's membrane. (a) High FAP expression in the superficial stroma (black arrow) and posterior stroma (red arrow). (b) Vimentin expression in the superficial stroma (black arrow) and posterior stroma (red arrow). (c) *α*-SMA expression in the superficial stroma (black arrow). (d) Negative controls in the absence of primary antibodies. Scale bar: 50 *μ*m.

Eight keratoconic corneal buttons harvested from the 8 advanced keratoconus patients with superficial stromal scarring underwent immunohistochemistry staining, and the scar regions expressed high immunogenicity of fibroblast activation proteins (FAP), vimentin, and *α*-smooth muscle actin (*α*-SMA) (Figures 5 and 6). Decentralized expression of FAP and vimentin was visible throughout the corneas of the patients with keratoconus, and in the superficial stroma, excessive expression of FAP and vimentin was observed (Figure 5). The significance of the *α*-SMA expression was that it was the major morphological characteristic of the myofibroblasts; however, there was high expression of *α*-SMA only in the superficial stroma (Figures 5 and 6).

## 4. Discussion

Superficial stromal scarring is usually observed in advanced keratoconus, and it affects vision severely, increases irregular astigmatism, and even leads to the failure of treatment with rigid gas permeable contact lenses [14]. According to previous pathologic studies, tenascin, laminin-5, perlecan, and type VII collagen are expressed to a greater extent in clinically and histologically scarred keratoconus [6], but there have been few investigations regarding the mechanism of superficial stromal scarring [5, 7]. With the help of in vivo confocal microscopy, we performed this study to investigate the consecutive microstructural changes in keratoconus and the potential mechanism for superficial stroma scarring in patients with healthy corneas, mild-to-moderate keratoconus, and advanced keratoconus.

As keratoconus progresses, keratocytes undergo significant changes. A keratocyte is a pinacocyte located in the gap of the parallel arrangement lamellae and is responsible for the synthesis and maintenance of the collagen fibrils and extracellular matrix. As an inactive mesenchymal cell, keratocytes usually preform a quiescent phenotype without abnormal stimulation, so the reflective and sharply demarcated cell nuclei of keratocytes are visible by scanning with in vivo confocal microscopy. In several confocal microscopy studies, the keratocyte density in patients with keratoconus was found to be significantly lower than in the normal controls, and decreases in anterior stromal keratocytes correlated with indices of disease severity [8, 10, 15]. Our study had different findings compared with previous research however. We found no difference in the density of sub-Bowman's stromal keratocytes between patients with mild-to-moderate keratoconus and the controls, but there was significant difference in the morphologic changes of sub-Bowman's stromal keratocytes, branched cell nuclei, and increased cytoplasm processes, indicating that these keratocytes had been activated. Contrary to quiescent keratocytes, the active keratocyte nuclei were less readily identified individually. The activated appearance of keratocytes suggested that an undetermined damage may stimulate fibrocytes to translate into fibroblasts and induce the mitosis of fibroblasts.

Previous studies suggested that the decrease in keratocytes and the imbalance of keratocyte synthesis in keratoconus resulted in a loss of stroma and a decrease in corneal biomechanics [8–10, 16]. Keratoconic cone may be related to an undetermined abnormality in corneal mechanical properties [17], such as corneal thinning and ectasia, caused by the loss of stromal lamellae [3, 18]. Through cohesive tensile strength testing, Dawson et al. found that the anterior 40% of the central corneal stroma was the strongest region of a normal cornea, and the posterior 20% was the weakest region [19]. Therefore, we conclude that the posterior cornea is the weakest region and may endure much smaller tension than anterior cornea. Clinically, the outstanding manifestation of mild keratoconus is that the posterior corneal surface often protrudes forward significantly more than the anterior corneal surface when viewed using corneal topography [20]. In our study, wide alternating dark and light bands were first detected in the posterior stroma of patients with mild-to-moderate keratoconus using confocal microscopy and were finally detected in both the anterior and posterior stroma of patients with advanced keratoconus. These findings may be related to the corneal biomechanic distribution and significant reduction in corneal biomechanics in advanced keratoconus. As reported previously, alternating dark and light bands represented folds of stromal lamellae [21], so alterations in the location and stage of the bands' distribution support the assertion that the abnormalities of the biomechanical properties first occur in the posterior stroma.

Meek et al. [22], through synchrotron X-ray scattering, detected systematic realignment of fibrils. This indicated a high degree of inter- and probably intralamellar displacement and slippage, which may be promoted by a loss of cohesive forces and mechanical failure and lead to thinning of the central cornea and associated changes in corneal curvature. Together with the protrusion of the posterior corneal surface, the anterior collagen withstands increasing tension, and this abnormal tension may lead to the slippage, folding, or even rupture of the anterior collagen lamellae. We hypothesize that this damage to the anterior collagen lamellae could cause the activation of keratocytes, and that is closely related to the formation of superficial stromal scarring. The collagen fibers produced by keratocytes were not visible with in vivo confocal microscopy, so we were unable to detect direct evidence of the slippage or rupture of the anterior collagen lamellae. Nevertheless, one important finding about the alteration of SNP in our study indirectly supported our hypothesis. We found that the nerve fibers had a thickened, prominent appearance, such as excessive branching and curling, and were even interrupted, which may be attributable to alterations in the corneal biomechanics in keratoconus, such as folds and rupturing of the stromal lamellae. Parissi et al. [23] reported that keratoconus was characterized by a progressive decreasing nerve density and altered nerve morphology, despite CXL treatment. In addition, it was reported that the anterior collagen lamellae were disturbed as observed by histopathology and X-ray scattering techniques [4, 17, 24]. The slippage of the lamellae, especially the anterior stromal lamellae, may stimulate the quiescent keratocytes to change and activate fibroblasts, thus corresponding with the higher reflective image of sub-Bowman's keratocytes in keratoconus. In this progression, mechanical failure stimulation may be a key regulator in keratocyte activation.

Changes in collagen fibers and lamellae can promote the transformation of quiescent keratocytes into fibroblasts and myofibroblasts, a repair phenotype of keratocytes [25]. To verify whether the keratocytes had been activated in accordance with our hypothesis, we evaluated the properties of fibrosis in scarred keratoconus using immunohistochemistry. Vimentin and FAP provide signs of keratocyte activation, and *α*-SMA is a sign of scar formation. We found that *α*-SMA, vimentin, and FAP were prominently expressed, with *α*-SMA only being highly expressed in the superficial stroma. These findings indicated that the keratocytes in keratoconus had been activated widely, and the formation of fibrosis was derived from myofibroblasts in the superficial stroma. Activated keratocytes may result in abnormal remodeling in keratoconus, contributing to the formation of superficial stromal scarring in advanced keratoconus.

The current study involved different stages of keratoconus, so we had an approximate horizon of the microstructure alterations in the development of keratoconus, except in patients who wear contact lenses. The semiautomated cell and nerve counter may have had unspecified measurement errors, which could be minimized with repeated and single-blind measurements. We observed keratocyte activation in keratoconus and verified it via the histopathological examination, but we had no idea of the initiation factor of keratocyte activation. A previous study [7] reported that growth factors from the epithelium may induce a fibrotic wound healing response following the rupture of Bowman's layer. To exclude the disturbance of epithelial-stromal interactions, longitudinal investigations on keratocyte functions under mechanical stimulation in keratoconus are needed, and these may reveal more insights into this putative association.

## 5. Conclusion

Abnormal microstructures detected in the keratoconic cone showed the slippage, folding, and even rupture of the anterior lamellae and a decrease in biomechanical strength in the development of keratoconus. All these abnormal alterations further encourage keratocyte activation and converting into fibroblasts, and even myofibroblasts, leading to the abnormal remodeling of ECM and the presence of superficial stromal scarring.

## Figures and Tables

**Table 1:** The classification of keratoconus.

Stage	Characteristics
Mild-to-moderate keratoconus	Eccentric corneal steepening
	Induced myopia and/or astigmatism ≤ 8 D
	Corneal radii ≤ 53 D
	Vogt striae, no scars
	Corneal thickness ≥ 400 *μ*m

Advanced keratoconus	Induced myopia and/or astigmatism > 8 D, even refraction not measurable
	Corneal radii > 53 D
	Corneal thickness < 400 *μ*m
	No acute corneal hydrops or perforation

**Table 2:** Demographic and clinical data.

	Control	Mild-to-moderate	Advanced	*P* value
Number of eyes	20	35	35	
Gender (male/female)	10/10	20/15	19/16	>0.05^a^
Age, years	23.2 ± 2.5	21.0 ± 4.2	21.5 ± 4.1	0.129^b^
CCT, *μ*m	553.3 ± 23.4	505.8 ± 25.1	390.6 ± 72.4	<0.001^c^
Cornea front				
K1, D	42.0 ± 1.3	43.7 ± 2.2	55.5 ± 7.4	<0.001^c^
K2, D	43.4 ± 1.3	45.3 ± 2.1	62.3 ± 8.8	<0.001^c^
Km, D	42.7 ± 1.3	44.5 ± 2.0	58.6 ± 7.8	<0.001^c^
Cornea back				
K1, D	−6.0 ± 0.2	−6.3 ± 0.4	−8.8 ± 1.8	<0.001^c^
K2, D	−6.4 ± 0.2	−6.9 ± 0.5	−10.1 ± 2.1	<0.001^c^
Km, D	−6.2 ± 0.2	−6.6 ± 0.4	−9.4 ± 1.8	<0.001^c^
KPD	1.2 ± 0.16	1.6 ± 0.5	4.3 ± 2.1	<0.001^c^

CCT: central corneal thickness; D: diopter; K: keratometry; KPD: keratometric power deviation.

^a^χ^2^ test; ^b^one-way ANOVA test; ^c^Kruskal-Wallis test.

**Table 3:** The density of the keratocytes and SNP.

	Controls	Mild-to-moderate	Advanced	*F*	*P* value
SBS keratocytes (cells/mm^2^)	608.9 ± 102.1	610.2 ± 149.3	508.4 ± 137.0	5.198	0.008^a^
*P* value		1.000^b^	0.036^c^		
AS keratocytes (cells/mm^2^)	409.9 ± 55.9	365.1 ± 47.6	301.4 ± 50.8	22.734	<0.001^a^
*P* value		0.002^b^	<0.001^c^		
PS keratocytes (cells/mm^2^)	374.5 ± 36.2	322.3 ± 41.3	254.3 ± 58.7	31.302	<0.001^a^
*P* value		0.003^b^	<0.001^c^		
SNP density, mm/mm^2^	15.9 ± 3.8	13.4 ± 3.9	8.5 ± 3.9	18.922	<0.001^a^
*P* value		0.284^b^	<0.001^c^		

SBS: sub-Bowman's stromal; AS: anterior stromal; PS: posterior stromal; SNP: subbasal nerve plexus.

^a^One-way ANOVA test; ^b^Bonferroni test between controls and mild-to-moderate keratoconus.

^c^Bonferroni test between controls and advanced keratoconus.

## References

[1] Rabinowitz Y. S. (1998). Keratoconus. *Survey of Ophthalmology*.

[2] Krachmer J. H., Feder R. S., Belin M. W. (1984). Keratoconus and related noninflammatory corneal thinning disorders. *Survey of Ophthalmology*.

[3] Mathew J. H., Goosey J. D., Bergmanson J. P. G. (2011). Quantified histopathology of the keratoconic cornea. *Optometry and Vision Science*.

[4] Cheung I. M. Y., McGhee C. N. J., Sherwin T. (2013). A new perspective on the pathobiology of keratoconus: interplay of stromal wound healing and reactive species-associated processes. *Clinical & Experimental Optometry*.

[5] Zhou L., Yue B. Y. J. T., Twining S. S., Sugar J., Feder R. S. (1996). Expression of wound healing and stress-related proteins in keratoconus corneas. *Current Eye Research*.

[6] Kenney M. C., Nesburn A. B., Burgeson R. E., Butkowski R. J., Ljubimov A. V. (1997). Abnormalities of the extracellular matrix in keratoconus corneas. *Cornea*.

[7] Tuori A., Virtanen I., Aine E., Uusitalo H. (1997). The expression of tenascin and fibronectin in keratoconus, scarred and normal human cornea. *Graefe's Archive for Clinical and Experimental Ophthalmology*.

[8] Ku J. Y. F., Niederer R. L., Patel D. V., Sherwin T., McGhee C. N. J. (2008). Laser scanning in vivo confocal analysis of keratocyte density in keratoconus. *Ophthalmology*.

[9] Hollingsworth J. G., Efron N., Tullo A. B. (2005). In vivo corneal confocal microscopy in keratoconus. *Ophthalmic & Physiological Optics*.

[10] Niederer R. L., Perumal D., Sherwin T., McGhee C. N. J. (2008). Laser scanning in vivo confocal microscopy reveals reduced innervation and reduction in cell density in all layers of the keratoconic cornea. *Investigative Ophthalmology & Visual Science*.

[11] Gore D. M., Shortt A. J., Allan B. D. (2013). New clinical pathways for keratoconus. *Eye*.

[12] Colin J., Velou S. (2003). Current surgical options for keratoconus. *Journal of Cataract and Refractive Surgery*.

[13] McMahon T. T., Szczotka-Flynn L., Barr J. T. (2006). A new method for grading the severity of keratoconus: the Keratoconus Severity Score (KSS). *Cornea*.

[14] Barr J. T., Wilson B. S., Gordon M. O. (2006). Estimation of the incidence and factors predictive of corneal scarring in the collaborative longitudinal evaluation of keratoconus (clek) study. *Cornea*.

[15] Ozgurhan E. B., Kara N., Yildirim A., Bozkurt E., Uslu H., Demirok A. (2013). Evaluation of corneal microstructure in keratoconus: a confocal microscopy study. *American Journal of Ophthalmology*.

[16] Hurmeric V., Sahin A., Ozge G., Bayer A. (2010). The relationship between corneal biomechanical properties and confocal microscopy findings in normal and keratoconic eyes. *Cornea*.

[17] Hayes S., Boote C., Tuft S. J., Quantock A. J., Meek K. M. (2007). A study of corneal thickness, shape and collagen organisation in keratoconus using videokeratography and X-ray scattering techniques. *Experimental Eye Research*.

[18] Morishige N., Wahlert A. J., Kenney M. C. (2007). Second-harmonic imaging microscopy of normal human and keratoconus cornea. *Investigative Ophthalmology & Visual Science*.

[19] Dawson D. G., Grossniklaus H. E., McCarey B. E., Edelhauser H. F. (2008). Biomechanical and wound healing characteristics of corneas after excimer laser keratorefractive surgery: is there a difference between advanced surface ablation and sub-Bowman's keratomileusis?. *Journal of Refractive Surgery*.

[20] Ishii R., Kamiya K., Igarashi A., Shimizu K., Utsumi Y., Kumanomido T. (2012). Correlation of corneal elevation with severity of keratoconus by means of anterior and posterior topographic analysis. *Cornea*.

[21] Hollingsworth J. G., Efron N. (2005). Observations of banding patterns (vogt striae) in keratoconus: a confocal microscopy study. *Cornea*.

[22] Meek K. M., Tuft S. J., Huang Y. (2005). Changes in collagen orientation and distribution in keratoconus corneas. *Investigative Ophthalmology & Visual Science*.

[23] Parissi M., Randjelovic S., Poletti E. (2015). Corneal nerve regeneration after collagen cross-linking treatment of keratoconus: a 5-year longitudinal study. *JAMA Ophthalmology*.

[24] Fullwood N. J., Tuft S. J., Malik N. S., Meek K. M., Ridgway A. E. A., Harrison R. J. (1992). Synchrotron x-ray diffraction studies of keratoconus corneal stroma. *Investigative Ophthalmology & Visual Science*.

[25] Muthusubramaniam L., Peng L., Zaitseva T., Paukshto M., Martin G. R., Desai T. A. (2012). Collagen fibril diameter and alignment promote the quiescent keratocyte phenotype. *Journal of Biomedical Materials Research—Part A*.

